# Learning gain of an ATLS^®^-based interprofessional and multidisciplinary in-situ simulation training of trauma resuscitation

**DOI:** 10.1007/s00068-026-03146-z

**Published:** 2026-03-17

**Authors:** Silke Hammer, Johan F. Lock, Sarah König, Oliver Happel, Mila M. Paul

**Affiliations:** 1 Department of Diagnostic and Interventional Radiology, University Hospital Würzburg, Oberduerrbacher Str. 6, 97080 Wuerzburg, Germany; 2 Department of General, Visceral, Transplantation, Vascular and Pediatric Surgery, University Hospital Wuerzburg, Oberduerrbacher Str. 6, 97080 Wuerzburg, Germany; 3 Institute for Medical Teaching and Medical Education Research, University Hospital Wuerzburg, Josef-Schneider-Str. 2, 97080 Wuerzburg, Germany; 4 Department of Anesthesiology, Intensive Care, Emergency and Pain Medicine, University Hospital Wuerzburg, Oberduerrbacher Str. 6, 97080 Wuerzburg, Germany; 5 Department of Trauma, Hand, Plastic and Reconstructive Surgery, University Hospital Wuerzburg, Oberduerrbacher Str. 6, 97080 Wuerzburg, Germany

**Keywords:** CRM, Simulation, Team training, Polytrauma, ATLS^®^, Factor analysis

## Abstract

**Purpose:**

Team performance in polytrauma management determines patient outcome and is crucially shaped by Crew Resource Management (CRM). This study explored patterns of self-perceived learning following an interdisciplinary, interprofessional, in-situ, simulation- and ATLS^®^-based trauma team training with a focus on CRM principles.

**Methods:**

We conducted a retrospective analysis of routine educational data collected immediately after 36 training sessions (03/2022–11/2023) including 238 participants at a single Level I trauma center. Participants completed a retrospective pre-test/post-test self-assessment questionnaire. Exploratory analysis, including EFA, were used to descriptively structure self-perceived learning patterns and subgroup differences.

**Results:**

Participants came from anesthesiology, surgery and radiology in equal proportions and differed in working experience, professional role, and exposure to polytrauma management. Exploratory factor analysis identified three descriptive dimensions of self-assessed CRM-related concepts: (i) personal operational competence, (ii) team communication, and (iii) decision making. Patterns of self-perceived change varied by experience level, with larger reported gains in personal operational competence among less experienced participants.

**Conclusion:**

This exploratory analysis describes patterns of self-perceived learning following in-situ trauma team simulation training. The findings should be interpreted as subjective learning perceptions at Kirkpatrick level 2 rather than evidence of long-term behavioral change. Self-perceived CRM-related competencies were enhanced in all professions and subspecialties, regardless of prior experience or trauma exposure. Future longitudinal and objective assessments are needed to evaluate transfer to practice and sustainability.

**Supplementary Information:**

The online version contains supplementary material available at 10.1007/s00068-026-03146-z.

## Introduction

Resuscitation and management of polytrauma patients involve complex and time-critical tasks, where survival depends on coordinated team performance. Worldwide, many organizations have formed multi-professional healthcare teams to manage critically ill patients, which can improve outcomes of severely injured patients [1]. In line with typical clinical practice, communication within these teams represents a unique challenge as they are frequently assembled spontaneously (‘ad-hoc’) and on short notice due to structural constraints. Individual members of the trauma team arise from distinct subspecialties such as trauma surgery, general surgery, anesthesia, radiology, and others. Whilst each member may have received a specific training in their respective disciplines, systematic preparation for interprofessional team performance and communication under acute stress is often lacking. Recent work has emphasized that these challenges are not primarily due to individual lack of expertise but rather to structural and organizational characteristics of interprofessional trauma teams, highlighting the need for targeted interprofessional training approaches [2].

For years, there has been a recommendation to provide training for healthcare professionals in teamwork and other non-technical skills [3]. Moreover, growing scientific evidence on Crew Resource Management (CRM) training effectiveness in medicine has globally spread this training principle in the past decades [4]. Recent systematic reviews further confirmed the effectiveness of simulation-based trauma team training in improving both technical and non-technical skills [5–8]. CRM, originally derived from the aviation industry, has demonstrated positive effects on teamwork and patient safety [9].

As one of 23 Level I trauma centers in Bavaria, Germany, the University Hospital in Wuerzburg launched an interdisciplinary and interprofessional in-situ team training (iSRST) based on ATLS^®^ (Advanced Trauma Life Support) principles and simulation scenarios in March 2022. Since then, trainings have taken place twice a year. It was designed to reflect the challenges in multidisciplinary, interprofessional and interpersonal collaboration. The educational concept consists of trauma patient scenarios and includes a theoretical introduction, two in-situ simulations followed by structured debriefings within a 4-hour program. Training concept and implementation received high acceptance and overall satisfaction [10]. To evaluate training effects, we applied Kirkpatrick’s framework of educational evaluation [11]. Since our design relied on self-assessed retrospective questionnaires, the analysis corresponds primarily to level 2 (‘Learning’). While self-assessment may only weakly correlate with objectively measured performance [12, 13] and post-intervention self-assessments almost invariably show improvement following educational exposure [14, 15], it is frequently used for feasibility reasons and to capture learner perceptions, particularly for cognitive and attitudinal outcomes that are not directly observable.

We hypothesized that participation would improve self-assessed perceived learning gain in defined CRM principles. However, it remains unclear whether different professional subgroups benefit equally, as transfer to real-life trauma care may depend on prior experience and role [1]. Therefore, this study aimed to identify underlying CRM principles in participant’s learning gains and to explore subgroup-specific differences. We formulated the following research questions:

- Which CRM-related competencies can be identified as underlying constructs in the self-assessed learning gains of participants in ATLS^®^-based in-situ trauma team training?
- Do different subgroups (profession, role, level of experience) benefit differently from the training in terms of CRM competencies?
- What implications arise for the curricular integration of interprofessional simulation trainings in trauma care?

## Methods

### Structure of the team training

The iSRST concept was developed by an interdisciplinary team consisting of physicians from the Departments of Trauma Surgery, General Surgery, Anesthesiology, and the Institute of Radiology, in collaboration with the simulation team of the Department of Anesthesiology, Intensive Care, Emergency and Pain Medicine at the University Hospital of Wuerzburg [16]. For voluntary participation, physicians from the mentioned departments and nurses from emergency department and anesthesia, as well as radiologic technologists (RT) were invited. This recruitment strategy ensured that training groups closely mirrored the real-life composition of ad-hoc trauma teams. The trainings were accompanied by audio and video recordings (SIMStation GmbH, Vienna, Austria) used to support structured debriefings. In the implementation of the trauma patient scenarios, a simulation phantom (Resusci Anne Simulator, Laerdal Medical AS, Stavanger, Norway) as well as standard non-sterile consumables simulated materials (e.g., mock blood products) were utilized. For radiological assessment, anonymized ultrasound and computed tomography (CT) images from actual clinical cases were provided. Each training session followed a standardized scheme: interactive team discussions regarding ATLS^®^ and CRM-principles followed by a phase to ensure participants were familiar with the technical environment (‘familiarization’). Next, training groups were engaged in two simulation scenarios starting with the patient’s announcement, following the standard algorithm for polytrauma care in the in-situ environment. Details of all simulation scenarios used in the years 2022 and 2023 including ATLS^®^-relevant decision points, are provided in Supplementary Table 1.

Scenarios were followed by structured debriefings using video and audio recordings. The training concluded with an oral feedback round. Finally, participants were asked to voluntarily and anonymously answer the questionnaires distributed at the end of each training session.

### Questionnaires

Data collection was conducted anonymously and on a voluntary basis. The initial questionnaire included demographic data and items on course satisfaction. In November 2023, the instrument was revised to additionally assess self-perceived learning progress regarding predefined learning objectives. The complete questionnaire, including the main question categories with scale values and items, is available Supplementary Table 2.

The subgroup analysis was conducted based on the demographic data regarding specialty (Surgery, Anesthesiology, Radiology), employment status (physician and non-physician positions), and work experience. In terms of work experience, the following groups were classified as ‘experts’: specialist physicians and assistants with more than 5 years of experience, physicians with advanced training and ATLS^®^ certification, and physicians with at least weekly trauma care practice and 3–5 years of experience. The remaining participants were labeled as ‘providers’. Since this study concentrated on the self-assessed retrospective pre-test/ post-test agreements to predefined CRM-concepts, Box 1 displays the original German wording of these items.

**Box 1:** Modified CRM main principles for trauma care

1. I am familiar with my work environment in the trauma bay.
2. I feel like an active member of the team.
3. I can voice my concerns at any time.
4. My opinion is heard.
5. The workflows in the trauma bay are clear to me.
6. The task distribution within the trauma team is clear to me.
7. I can ask for help at any time.
8. I recognize fixation errors and can avoid them.
9. I consider the implementation of short team briefings (10-for-10) to be relevant.
10. I discard regular reevaluations according to the ABCDE approach to be meaningful.

Evaluation items were adapted from CRM principles outlined by Rall and Gaba [17]. Items were streamlined and to focus on team-related attitudes rather than practical instructions (e.g., the original item ‘use mnemonics and look up’ was omitted).

### Statistical analysis

Analyses were conducted using descriptive statistics, including mean (M), minimum (Min), maximum (Max), standard deviation (SD), and skewness (Skew) of all questionnaire items. Mean individual and group learning gains were firstly calculated as a composite sum score (‘CRM-score’). We subjected the retrospective pretest assessments for CRM-criteria to an exploratory factor analysis (EFA) using maximum likelihood estimation and promax rotation to identify underlying dimensions of CRM-related constructs. Criteria for adequacy were *p* < 0.05 in Bartlett’s test of sphericity and a KMO coefficient > 0.50. The KMO coefficient indicated a high level of sampling adequacy (0.84), and Bartlett’s test of sphericity yielded a significant result (χ2 = 1158.707; df = 45; *p* < 0.001). Internal Consistency was evaluated using Cronbach’s alpha (α), where a value above 0.7 was deemed good, while between 0.6 and 0.7 was considered acceptable. Between-groups differences were analyzed using ANOVA and Welch-test. Pearson-correlation coefficients were computed to examine associations between items.

### Ethics approval

The local institutional review and ethics board deemed the project not to constitute medical or epidemiological research on human subjects and therefore applied a simplified assessment protocol. The study design was reviewed by the Ethics Committee of the University of Wuerzburg, which confirmed that no formal consultation was required in accordance with § 15 of the Professional Code of Physicians (Decision No. 2022101101). Survey data from the questionnaires were retrieved anonymously using the EvaSys^®^ platform (Lueneburg, Germany). Participation was voluntary, and data were processed and stored in compliance with local data protection regulations and the EU General Data Protection Regulatio. Audio and video recordings served exclusively to support structured debriefings during training and were not analyzed for research purpose.

## Results

### Demographics

**Fig. 1:**
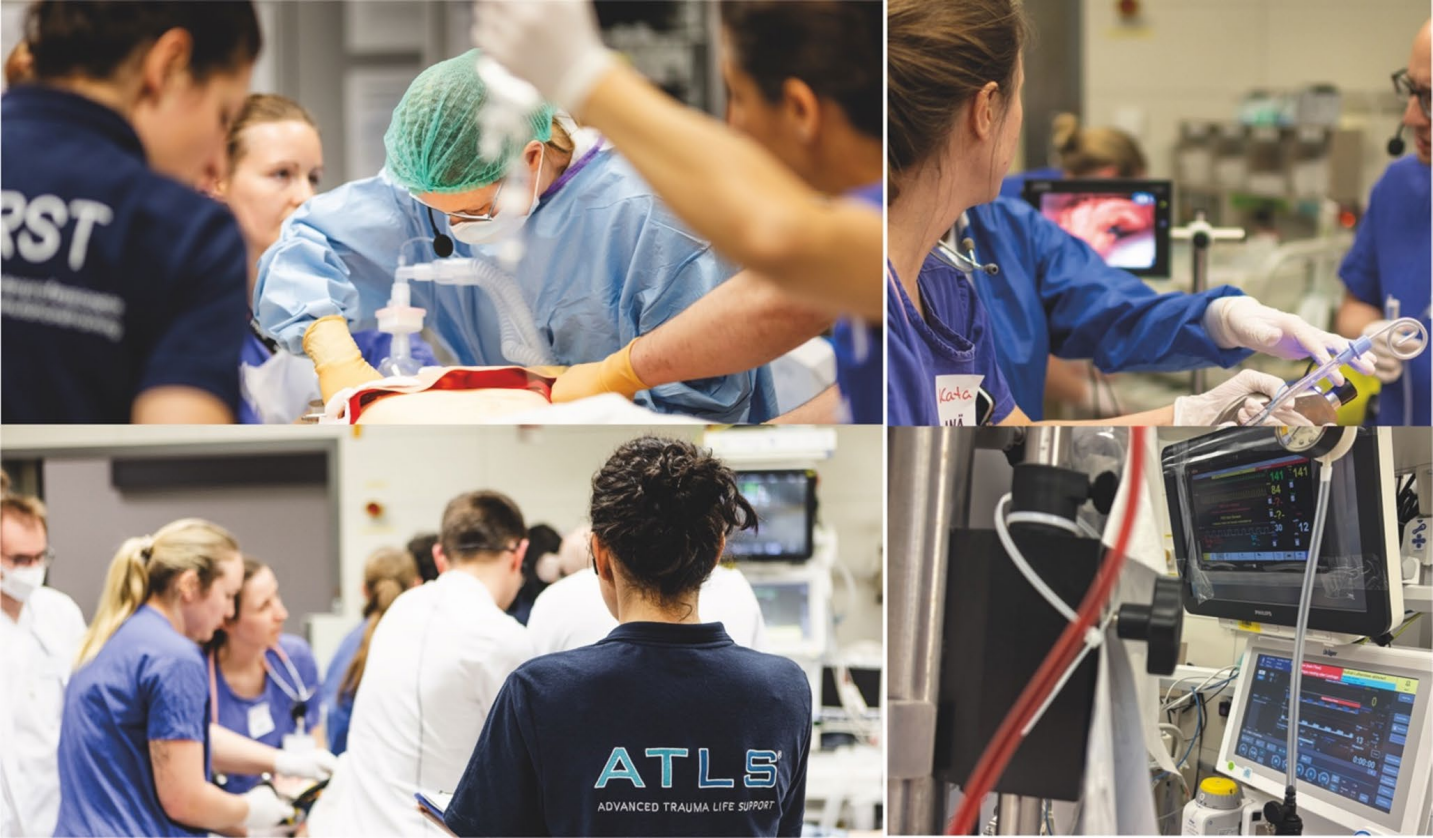
Original snapshots from training scenarios. Participants were trained in performing technical and non-technical skills in realistic, ATLS^®^-based polytrauma simulation cases

In 2022 and 2023, a total of 238 doctors, nurses and RTs participated in the interdisciplinary team training (Fig. 1; Table 1). The response rate for the questionnaires distributed at the end of each training session was 100%. Thus, 238 participants provided responses regarding their self-assessed learning gains in relation to CRM principles. Of these, 230 questionnaires were fully completed. During the period of this study, eight trauma scenarios were performed. Training groups consisted of eight individuals on average. Characteristics of training participants are listed in Table 1. There was an even distribution between females (53%) and males (47%). According to in-house standards, teams were built by staff from Anesthesiology, Trauma and General Surgery, and Radiology. Nurses from surgery departments are listed as emergency department staff, radiology technicians (RT) were included in the subgroup of radiology (explaining the high number of participants, *n* = 28.1%). Nearly half of the participants were resident doctors, only 10.6% were specialists (senior doctors or consultants) and only 2% had senior positions (e.g., deputy head or director). 39.6% of all participants were trainee nurses, registered nurses or RTs. In terms of working experience, participants with 3–5 years prevailed (38.1%). About 50% of the study group stated that they participated at least weekly in polytrauma management (Table 1).

**Table 1:** Characteristics of the study group. The table highlights demographic characteristics of all training participants in 2022 and 2023 constituting the study group. Absolute numbers and percentages are reported for age groups, gender, department, professional role, working experience and frequency of participation in polytrauma care

	Subgroup	*n* = 238	*n* [%]
Age [years]			
	< 30	84	35.4
	31–40	111	46.8
	41–50	22	9.3
	51–60	16	6.8
	> 60	4	1.7
Gender			
	Female	125	52.7
	Male	112	47.3
Department			
	Anesthesiology	84	35.7
	General surgery	27	11.5
	Trauma surgery	28	11.9
	Radiology	66	28.1
	Emergency department staff	28	11.9
Professional role			
	Resident physician	144	47.8
	Specialist physician	32	10.6
	Senior medical position	6	2.0
	Trainee nurse	10	3.3
	Registered nurse	68	22.6
	Radiologic technician	41	13.7
Working experience			
	< 1 year	25	7.9
	1–2 years	38	12.1
	3–5 years	120	38.1
	6–9 years	44	14.0
	10–19 years	55	17.5
	≥ 20 years	33	10.5
Frequency of participation in polytrauma care			
	Rarely	8	3.4
	Occasionally	60	25.8
	Monthly	29	12.4
	Weekly	96	41.2
	Daily	40	17.2

**Table 2:** Factor composition based on the exploratory factor analysis (EFA) for self-reported pre-test values. Cross-loadings with lower coefficients are not shown

Personal operational competence	Team communication	Decision making
1. I am familiar with my work environment in the trauma bay.	3. I can voice my concerns at any time.	9. I consider conducting short team meetings (10-for-10) relevant.
2. I feel like an active member of the team.	4. My opinion is heard.	10. I discard regular reevaluations according to the ABCDE approach to be meaningful.
5. The workflow in the trauma bay is clear to me.	7. I can ask for help anytime.	
6. The task distribution within the trauma team is clear to me.		
8. I recognize fixation errors and can avoid them.		

### Analysis of self-estimated learning gain

Between-group comparisons were conducted regarding the sum retrospective learning gains for all items, defined as the ‘CRM score’ difference (Δ = post – pre). Taking the ten items on the five-point Likert-scale into consideration, the highest theoretically attainable CRM-Score for an individual was 40. In the whole cohort, learning gain scores ranged between minimum − 3 and maximum 24 with a low mean value of ± SD of 4.95 ± 4.80 (12.4% of the possible 40-point gain). The average sum pre-test CRM-score was high at 37.94 points (76% of 50; SD = 6.46). In the post-test, the entire group showed even a higher average of 42.85 points (86% of 50; SD = 4.07). The cumulative retrospective learning gain was higher for female participants (5.16 ± 5.04) than for males (4.75 ± 4.37; F _[23/205]_ = 1.66; *p* < 0.05). However, there was no difference among the groups based on department, professional role, working experience, and frequency of participation in polytrauma care (Supplementary Table 3). The effect sizes of the mentioned subgroups were consistently high and varied between 0.08 (department) and 0.16 (gender). Regarding the higher-level categories mentioned above, there was a higher self-assessed learning gain for providers (6.02 ± 5.69) than experts (4.13 ± 3.59; F _[1/228]_ = 9.44); *p* < 0.01). Regarding training experience, no difference was found between the groups in terms of the CRM score (F _[1/,72]_ = 0.67; *p* = 0.42).

### Subgroup analysis of underlying CRM principles

**Fig. 2:**
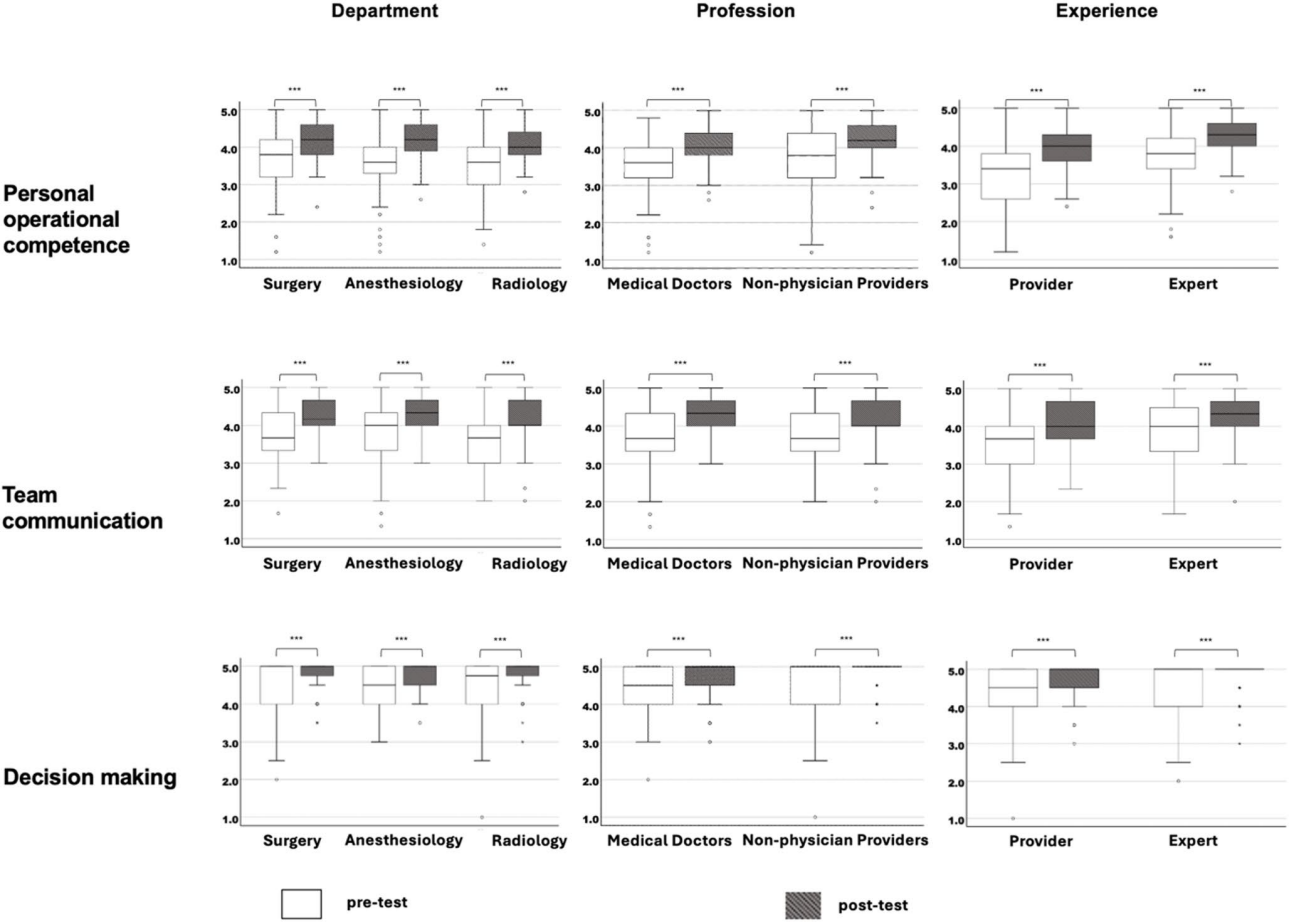
Pre-test/ post-test differences in retrospective self-assessed competences by department, experience, and profession. ***p < 0.001, significant between-group differences not visualized. White and grey boxes represent values before and after the training, respectively (pre-test/ post-test). In box plots, horizontal lines represent median, boxes quartiles and whiskers 10th and 90th percentiles. Scatter plots show individual outliers

Based on the criteria outlined in the statistical analysis section, a three-factor solution was found to offer the most suitable fit and demonstrated good internal consistency. We describe these three factors as follows (Fig. 2):

1. *Personal operational competence*: the self-assessed competence within the trauma bay working environment including knowledge about structural resources and role distribution and processes, number of items = 5.
2. *Team communication*: the knowledge that personal opinions are heard within the team and can be freely expressed without social pressure, number of items = 3.
3. *Decision making*: the awareness that the situation needs to be dynamically reevaluated and team time-outs are necessary, number of items = 2.

The corresponding factor loadings are detailed in the Supplementary Material (Supplementary Table 4). In total, the three factors accounted for 52% of the item variance. Moderate Pearson correlation coefficients (0.31–0.54; *p* < 0.001) indicate a strong discriminative ability of the scales. Cronbach’s α for the three factors was 0.68, representing acceptable internal consistency without evidence of redundancy.

Mean sum pre-test and post-test scores for the three factors revealed no significant differences across the departments. Regarding prior working experience, significant differences were found for the first two factors in pre-test and post-test values (*p* < 0.001). The professional role did not influence pre-test and post-test ratings of any factor. Learning gains (Δ = post – pre) were computed for each CRM factor (Fig. 2). Between group analyses demonstrated no significant difference across departments (*p* = 0.848). Prior simulation training experience did not affect learning gains on the factors (*p* > 0.05). When groups were categorized by professional experience (experts vs. providers), providers demonstrated larger gains in Personal operational competence (F _[1/231]_ = 9.24; *p* < 0.01), whereas Team communication and Decision making did not differ between groups (all *p* > 0.05). No differences by professional affiliation (physicians vs. non-physician participants) were observed for any factor (all *p* > 0.05). Paired t-tests confirmed significant pre–post improvements across all subgroups and factors (all *p* < 0.001, Fig. 2).

## Discussion

For trauma resuscitation and management of polytrauma patients, interprofessional communication and teamwork in the trauma bay are critical factors that largely contribute to sentinel events. Simulation-based team trainings is therefore a plausible approach to practice both technical and non-technical skills under realistic conditions. This is in line with findings from recent systematic reviews and meta-analyses demonstrating that simulation-based training improves team performance and human factor skills across diverse healthcare settings [7, 18]. In this context, it improves both technical and non-technical skills [19, 20]. Whereas the literature strongly supports the benefits of interprofessional CRM trainings in medical teams regarding communication and coordination [7, 21, 22], there is limited evidence on which CRM dimensions benefit for which subgroups. In this study, we evaluated an individually designed simulation-based interdisciplinary trauma team training at a Level I trauma center in Germany based on ATLS^®^ principles. Our aim was to characterize the structure of perceived learning gains across CRM dimensions and to explore subgroup differences by role, profession, and prior experience to inform instructional design. Recent studies have addressed particularly needs for team training in ad-hoc teams and report performance gains even without stable team constellations [2, 23]. In line with Kirkpatrick’s framework, our evaluation corresponds to level 2 (‘Learning’) and relies on retrospective self-assessed knowledge gain [11]. Retrospective self-assessments may be affected by recall bias as well as social desirability or demand characteristics. These influences are well described in the methodological literature on retrospective self-evaluation and may impact the magnitude of self-reported learning gains [24, 25]. While medical education literature suggests a weak correlation between self-assessment and objective performance measures [12], self-reports are pragmatic and minimize testing reactivity [13].

The interpretation of learning gains in educational studies depends on the level of learning being assessed. Beyond factual knowledge or observable performance, learning has been conceptualized as a process of conceptual and metacognitive recalibration, particularly in complex domains such as emergency care. From this perspective, changes in self-assessed competence may reflect meaningful shifts in participants’ internal frames of reference, awareness of task complexity, and understanding of professional requirements rather than mere response artifacts. Such recalibration has been described as an integral component of learning, even when objective performance gains are not immediately measurable. Thus, perceived subjective learning gains measured in this study should not be interpreted solely as increases in factual knowledge, but as indicators of underlying changes in participants’ conceptual understanding of polytrauma care team competence. Due to the course concept involving voluntary participation, implementation of a structured summative assessment was not applicable. Given voluntary participation, summative performance testing was not feasible. The interpretation of the observed learning gains must also consider the high retrospective pre-test scores across several CRM-related dimensions, indicating a potential ceiling effect. Many participants reported substantial baseline familiarity with CRM concepts prior to the training, which inherently limits the headroom for measurable improvement and constrains the magnitude of post-test gains. Accordingly, the absolute learning gains observed were small relative to the overall scale range. While these differences reached statistical significance, their educational relevance should be interpreted with caution. Rather than reflecting substantial competence acquisition, the findings are best understood as modest shifts in self-perceived learning within an already highly trained cohort.

The EFA revealed a three-factor model comprising personal operational competence, team communication, and decision making. Internal consistency was acceptable, and inter-item correlations indicated related yet distinct. While the first factor (*personal operational competence*) primarily addressed technical aspects within the working environment, the other two factors focused on non-technical team interaction. Within this framework, the largest mean learning gains occurred in items related to operational/ technical knowledge, particularly among less experienced participants (‘providers’). In healthcare training, technical and non-technical skills are typically regarded as two distinct concepts, both of which are crucial in managing and preventing critical or adverse events [26, 27]. Non-technical skills are generally seen as cognitive and social skills, whereas technical skills involve the use of medical equipment and drugs, along with specific medical expertise [28].

The factors *team communication* and *decision making* showed highly significant gains across participant groups. The finding of significant knowledge gains across all demographic subgroups warrants careful interpretation. While this pattern may reflect a broadly effective training intervention, it may also be partially influenced by the retrospective pretest–posttest design employed. Retrospective self-assessment is known to be susceptible to response-shift bias, whereby participants recalibrate their understanding of the assessed constructs after exposure to the intervention, leading to lower retrospective ratings of baseline knowledge. This effect can result in consistent pre–post differences across subgroups, independent of demographic characteristics. At the same time, such recalibration has been described as a meaningful indicator of learning in complex educational settings, where participants’ conceptual understanding of the domain evolves through training. Consequently, the observed knowledge gains likely represent a combination of true learning effects and changes in participants’ internal frames of reference. The finding that experienced clinicians demonstrated improvements in decision-making underscores that routine clinical exposure alone does not fully address the cognitive and metacognitive components of expertise, which may be specifically targeted through structured simulation and debriefing.

The concept of team communication and decision-making touches upon the utilization of all available personnel resources without restriction, for example, due to existing hierarchies. Previous work in context of trauma teams support flatter structures [29, 30], allowing team members to interact and communicate on an equal footing with a high amount of psychological [31]. Regarding *team communication*, no subgroup differences by department, experience, or profession were observed pre-post, indicating that all groups benefit from the training intervention. Our findings confirm that simulation-based training within trauma teams enhance communication and performance irrespective of prior job-related experience, with particularly pronounced gains in personal operational competence (workplace/ process) among less experienced participants. Therefore, it is reasonable not only to include members at all levels of experience in the training, but also to design scenarios that explicitly address communication-related issues and reinforce flat hierarchies. Finally, the attitude that situations should be dynamically reevaluated and repeated team time-outs are necessary (summarized in *decision making*) yielded high pre- and post-test self-evaluated scores across all subgroups without between-group differences. Given the relatively small subgroup sizes, additional data collection may reveal a positive trend in this aspect with repeated training sessions.

A small but statistically significant difference in overall self-assessed learning gains was observed between female and male participants. This finding requires cautious interpretation. Previous research has consistently shown that self-assessment data may be influenced by gender-related differences in self-evaluation and response-shift effects. In particular, female participants have been reported to rate their baseline competencies more conservatively and to show greater recalibration following educational interventions, which may result in higher measured learning gains in retrospective pretest–posttest designs without necessarily indicating greater objective competence acquisition [12–14]. The present findings should be interpreted in light of the exploratory analytical approach adopted in this study. Both the factor analysis and the subsequent subgroup comparisons were conducted with the primary aim of describing patterns of perceived learning gains and generating hypotheses for future research, rather than providing confirmatory evidence. Accordingly, the identified factor structure represents a preliminary model that warrants validation in independent samples. Confirmatory testing of the dimensional structure and subgroup effects would require larger cohorts, prospective study designs, and the application of confirmatory factor analysis. Within this context, the current results provide an initial framework to inform instructional design and to guide the development of targeted simulation-based training interventions.

To date, it remains unclear whether self-reported pre-test values change among participants attending multiple training sessions over time.

In summary, the iSRST appears to improve subjective concepts regarding collaboration within the specified team structures. The training proves advantageous for all participating subgroups, regardless of their professional background, expertise or prior training experience. This is consistent with previous studies that both surgeons and anesthetists’ benefit from simulation-based training [32–34].

Nonetheless, there are specific limitations that need to be addressed. Firstly, our evaluation was designed to cover the second level of Kirkpatrick’s framework (‘Learning’). However, the levels 3 and 4 (corresponding to ‘Did the intervention result in a change of behavior?’ and ‘Did the intervention influence performance?’), still need to be evaluated in detail. Previous studies have reported longer time spent on trauma patients [35]. For this purpose, long-term data collection and statistical evaluation is warranted. Previous studies have addressed Kirkpatrick’s level 3 by comparing video-records of preintervention and repeated post-intervention simulations [36]. The present analysis was not intended to detect changes in global clinical endpoints (e.g., mortality, morbidity, and length of stay), which refer to level 4 by Kirkpatrick. Interpretation of these outcomes must incorporate additional context information, as they are often the result of a constellation of multiple factors and are less under the team’s direct control.

Secondly, the durability of effects after a single short session remains uncertain; mid- and long-term follow-up is needed. Some authors reported retained improvement in non-technical skills following one-day training sessions in short timespans of one or two months [37]. Comparable findings have also been demonstrated in trauma-focused ATLS^®^-based trainings [38] and in prehospital emergency simulation settings [39]. Thus, the iSRST needs to be mid- and long-term evaluated. In addition, repeated training sessions will likely be necessary to sustain improvement regarding the CRM-concepts. The optimal time frequency and duration require further investigation. Regarding our individual course concept, participants evaluated that a frequency of 1–2 trainings per year suited their expectations towards the training best. Thirdly, our data fully relied on subjective self-assessment. Evaluating self-assessed competencies and calculating retrospective learning gains may be limited indicators of actual knowledge gain. While retrospective pretest–posttest designs are limited in their ability to assess true longitudinal retention, they are particularly suited to complex educational interventions in which participants’ baseline self-assessments may change after training [40, 41]. In retrospective pretest-posttest designs, participants rate their status and retrospectively their prior status within the same measurement occasion, thereby using a common frame of reference and reducing bias introduced by changes in understanding of the construct over time. Moreover, recent reviews in medical education highlight the pragmatic value of retrospective and post-only assessment designs when traditional pretest administration is constrained by logistic or construct-clarity issues [42]. A meta-analysis on this topic indicates that while self-assessments are commonly used in literature for evaluation purposes, they may be imperfect and unreliable indicators of underlying true learning [13]. While self-assessment captures perceived competence, objective approaches such as knowledge testing, simulation-based performance assessment, observational rating scales, and workplace-based assessments are commonly used to evaluate competence beyond subjective measures.

In the present setting, retention assessment would have been methodologically challenging, as participants were frequently re-exposed to interdisciplinary team training or related clinical experiences, sometimes within short intervals. Such repeated exposure would likely confound delayed measurements and limit attribution of retention effects to the intervention itself. Future research on the training concept may address this limitation by incorporating delayed assessments while controlling for interim training exposure or by using objective performance-based retention measures.

Furthermore, there is a lack of consensus in the interpretation of self-assessments, sometimes treated as a facet of reactions (analogous Kirkpatrick’s level 1) and sometimes as an indicator of knowledge levels (analogous Kirkpatrick’s level 2) (‘Is Teacher Immediacy Actually Related to Student Cognitive Learning?’ [43]). Fourthly, our analysis used a purpose-designed individual questionnaire. Additional training aspects may not have been captured to the full extend. The single questionnaire precludes any conclusion regarding the sustainability of training effects. Finally, this was a single-center study conducted in a specific institutional context, which may limit the generalizability. Future multi-center trials with objective outcome measures and longitudinal follow-up are warranted to strengthen the evidence base.

## Conclusions

In this single-center study, an ATLS^®^-based, interprofessional in-situ trauma team training produced significant pre-post gains in CRM-related competencies across professions, experience and clinical role. Providers showed the largest improvement in personal operational competence, whereas team communication and decision-making were comparable across subgroups. These findings support embedding structured, simulation-based team trainings with structured debriefings as a routine component of quality management in trauma centers and as targeted scaffolding for early-career staff. Future work should determine durability and optimal training dose and evaluate behavioral (Kirkpatrick’s level 3) and patient/ process outcomes (level 4) using objective measures in multi-center, longitudinal designs.

## Supplementary Information

Below is the link to the electronic supplementary material.

Supplementary Material 1

## Data Availability

The authors confirm that the data supporting the findings of this study are available within this article. For further inquiries, please contact the corresponding author.
