# Supplementary material for: Learning gain of an ATLS®-based interprofessional and multidisciplinary in-situ simulation training of trauma resuscitation
Source: Eur J Trauma Emerg Surg. 2026 Mar 17;52(1):104. doi: 10.1007/s00068-026-03146-z (PMC12996380; doi:10.1007/s00068-026-03146-z)
Supplement: Supplementary file 1 — Supplementary Material 1 [file 68_2026_3146_MOESM1_ESM.docx]

**Supplementary Material**

**Supplementary Table 1. Design of training scenarios.**

Announcements including basic patient information, injury patterns and critical treatment decisions according to ATLS® are listed for all scenarios that took place from 03/2022 to 11/2023.

|  | **announcement** | **injury pattern** | **critical treatment decisions according to ATLS®** |
| --- | --- | --- | --- |
| **Scenario 1**  **03/2022** | 16 years ♀  hit by a horse | left thoracic trauma  ruptured spleen | **A:** airway management, endotracheal tube vs. supraglottic airway, spine immobilization  **B:** chest drain insertion  **C:** recognition and treatment of hemorrhagic shock  **D, E:** / |
| **Scenario 2**  **03/2022** | 58 years ♂︎  E-Bike accident | unstable pelvic ring fracture  multiple deep lacerations of left thigh | **A:** malposition of endotracheal tube in right main bronchus, spine immobilization  **B:** bad oxygenation due to tube misplacement  **C:** recognition and treatment of hemorrhagic shock, need to correct position of pelvic stabilization device, bleeding control  **D, E:** / |
| **Scenario 1**  **11/2022** | 62 years ♀  car accident | unstable pelvic ring fracture  open fractures of left foot  facial lacerations | **A:** airway management, spine immobilization  **B, D:** /  **C:** recognition and treatment of hemorrhagic shock, application of pelvic stabilization device  **E:** distracting injuries |
| **Scenario 2**  **11/2022** | 26 years ♂︎  motorcycle accident | left diaphragmatic rupture  left femur fracture  right humerus fracture  severe traumatic brain injury (TBI) | **A:** airway management, spine immobilization  **B:** impaired oxygenation due to herniated viscera  **C:** recognition and treatment of hemorrhagic shock  **D:** permissive hypotension vs. maintaining adequate cerebral perfusion pressure in TBI |
| **Scenario 1**  **03/2023** | 28 years ♀  car accident  22 weeks pregnant | rupture of uterine wall  right distal radius fracture | **A:** airway management, spine immobilization  **B:** /  **C:** recognition and treatment of hemorrhagic shock in a pregnant patient  **D, E:** / |
| **Scenario 2**  **03/2023** | 80 years ♂︎  motorcycle accident | left pneumothorax  pelvic ring fracture  left femur fracture  right leg nearly amputated below knee | **A:** airway management, spine immobilization  **B:** impaired oxygenation, chest drain insertion  **C:** recognition and treatment of hemorrhagic shock  **D:** /  **E:** hypothermia |
| **Scenario 1**  **11/2023** | 25 years ♂︎  car accident | multiple rib fractures  with hemothorax  mesenterial laceration  pelvic ring fracture  open left femur fracture  cardiac arrest at arrival | **A:** airway management, spine immobilization  **B:** chest drain insertion  **C:** recognition and treatment of hemorrhagic shock  **D, E:** /  treatment algorithm for traumatic cardiac arrest, discussion of non-invasive and invasive strategies |
| **Scenario 2**  **11/2023** | 74 years ♂︎  fall from height | TBI  multiple face and head lacerations  contusion of spine and pelvis | **A:** airway management, spine immobilization  **B:** /  **C:** impeding hemorrhagic shock (anticoagulants)  **D:** GCS 10  **E:** / |

**Supplementary Table 2**. **Outline of measures addressed in the questionnaire.**

| **main question category** | **definition** | **scale value and labels** |
| --- | --- | --- |
| **participant attributes** | gender, age, department, professional position, working experience, trauma center, frequency of participation in trauma care, training experience | 9 items |
| **personality traits** | Adapted to the ‘big five’ personality traits | 21 items on a 5-point Likert-scale  1=very inaccurate, 5=very accurate |
| **assessment of training quality** | presentation content, clinical relevance, implementation of scenarios, didactic quality, opportunity for practice, debriefing atmosphere  repetition interval | 11 items on a 5-point Likert-scale adapted to school grades  1=very good, 5=bad  single choice between ‘semi-annual’ and ‘never’ |
| **self-estimated pre- and post- retrospective learning gain regarding CRM principles** | familiarity with work environment, possibility to ask for help, feeling as a team-member, opportunity to express doubts, opinion is heard, procedures, task distribution, opportunity to ask for help any time, recognizing fixation errors, need for reevaluation | 10 items on a 5-point Likert-scale  1=very inaccurate, 5=very accurate |
| **institutional quality of trauma care** | preparation for disasters, training offers | 4 items on a 5-point Likert-scale  1=very inaccurate, 5=very accurate |
| **final assessment** | comments and critique | free text |

**Supplementary Table 3. Differences and group effect sizes in the ANOVA between the demographic groups for the summative retrospective learning gain.**

| **item** | **p-value** | **effect size** |
| --- | --- | --- |
| gender | 0.03 * | 0.16 |
| department | 0.79 | 0.08 |
| professional role | 0.46 | 0.10 |
| working experience | 0.20 | 0.12 |
| frequency of participation in polytrauma care | 0.08 | 0.14 |

**Supplementary Table 4. Exploratory factor analysis (EFA) for the items of self-reported pre-test values regarding CRM-criteria.**

Loadings between − 0.3 and 0.3 not displayed

|  | **Personal operational competence** | **Team communication** | **Decision making** | **mean** | **SD** |
| --- | --- | --- | --- | --- | --- |
| 1. I am familiar with my work environment in the ER. | 0.756 |  |  | 3.51 | 1.09 |
| 2. I feel like an active member of the team. | 0.610 | 0.447 |  | 3.78 | 0.99 |
| 5. The workflow in the ER is clear to me. | 0.838 |  |  | 3.71 | 1.06 |
| 6. The assignment of tasks within the trauma team is clear to me. | 0.753 |  |  | 3.63 | 1.06 |
| 8. I recognize fixation errors and can avoid them. | 0.516 |  |  | 3.29 | 0.76 |
| 3. I can express my concerns at any time. |  | 0.908 |  | 3.60 | 0.99 |
| 4. My opinion is heard. |  | 0.709 |  | 3.65 | 0.95 |
| 7. I can ask for help anytime. | 0.367 | 0.521 |  | 3.95 | 0.84 |
| 9. I consider conducting short team meetings (10-for-10) relevant. |  |  | 0.990 | 4.34 | 0.85 |
| 10. I discard regular reevaluations according to the ABCDE approach to be meaningful. |  |  | 0.623 | 4.50 | 0.71 |
